# What anabolic–androgenic steroids reveal about the limits of current harm reduction models

**DOI:** 10.1111/add.70395

**Published:** 2026-03-11

**Authors:** Timothy Piatkowski, Jason M. Nagata, Suzanne Nielsen

**Affiliations:** ^1^ Centre for Health Services Research and Centre for Community Health and Wellbeing, Faculty of Health, Medicine, and Behavioural Sciences The University of Queensland Brisbane Queensland Australia; ^2^ Queensland Injectors Voice for Advocacy and Action Sunshine Coast Queensland Australia; ^3^ Department of Pediatrics University of California San Francisco California USA; ^4^ Monash Addiction Research Centre, Eastern Health Clinical School Monash University Melbourne Victoria Australia

**Keywords:** anabolic–androgenic steroids, harm reduction, health services, image and performance enhancing drugs, injecting, route of administration

## Abstract

**Background:**

Harm reduction has largely been shaped by responses to psychoactive drug use where the most urgent harms are acute. These models focus on overdose, blood‐borne viruses, and rapid‐onset toxicity related harms. When applied wholesale to anabolic–androgenic steroids (AAS), they obscure the distinctive pharmacology, consumer typologies, and slow‐developing physiological risks that define people who use AAS.

**Argument:**

AAS use is often chronic, patterned, and long‐term. Harms are often cumulative and organ‐based rather than event‐based. Routes of administration carry different risk profiles, with oral formulations being more hepatotoxic and commonly falsified through mislabelling or adulteration than injectable products. Despite this, most health services position injecting as inherently higher risk, applying paradigms developed for opioids and stimulants that have not been adapted for AAS use. Using evidence across pharmacology, delivery routes, dependence trajectories, and consumer types, this paper argues for expanding harm reduction models to consider the unique needs of different populations of people who use drugs, including AAS. We draw critically on lessons from other substances as well as multiple existing approaches including structured dosing frameworks, and supply‐checking infrastructures, provide practical templates for expanding adaptation to AAS.

**Conclusions:**

To remain evidence‐based, harm reduction must evolve to be contextually relevant. We have used AAS as an example of this evolution, attempting to highlight cross‐substance learnings which integrate consumer‐focused tools, workforce development, and peer‐led support.

## INTRODUCTION

For more than three decades [1], harm reduction has evolved primarily in response to psychoactive drug use, particularly opioids and stimulants, where the most immediate threats are overdose, infection and blood‐borne viruses (BBVs) [2, 3]. This history has produced a durable set of assumptions about what *risk* and *harm* look like, and how they should be prevented: interventions are designed to manage acute, event‐based risks such as opioid overdose through sterile injecting, drug checking, supervised consumption rooms and safe supply initiatives [4, 5, 6, 7]. These approaches have saved countless lives. Yet when applied wholesale to other drug categories, they risk missing the fundamental differences in pharmacology, consumer trajectories and temporal patterns of harm.

Anabolic–androgenic steroids (AAS) represent a mismatch between substance‐specific harms and existing harm reduction models. Although commonly injected, AAS do not produce overdose in the conventional sense; their harms are less acute and more commonly cumulative and organ based, unfolding over years rather than hours [8, 9]. AAS use is typically structured, cyclical and goal‐oriented [10], yet public health responses have largely subsumed AAS consumers into services designed for people who inject psychoactive drugs, implicitly treating injection as the primary risk factor. This has produced service environments that prioritise viral prevention and sterile technique, while overlooking the distinct pharmacological and physiological risks faced by both early initiates and long‐term AAS consumers. Evidence indicates that AAS delivery routes—oral, intramuscular, subcutaneous and transdermal—carry distinct harm profiles [11]. Community AAS checking data show that oral formulations are more frequently falsified than injectables (40% vs 22%) [12]. While injectable AAS bypass hepatic metabolism and allow more stable serum concentrations [13], they require technical knowledge of dosing intervals and sterile administration. These pharmacological differences, alongside long‐term use, recognised AAS dependence [14] and widespread non‐medical consumption [15], highlight the need to broaden harm reduction frameworks. Therefore, this article argues for a reorientation of harm reduction to include AAS, towards more integrated models that consider harm across vectors of acuity, chronicity and route of administration.

## WHO USES WHAT, AND HOW: TYPOLOGIES AND DELIVERY CONTEXTS

The use of AAS has expanded well beyond its early association with elite sport to encompass a diverse and increasingly heterogeneous population. Global lifetime prevalence estimates suggest that 3%–6% of men and 1%–4% of women report some history of AAS use [16, 17], though rates are substantially higher among subgroups such as gym‐attending men, bodybuilders and fitness professionals [18], as well as cisgender gay and bisexual men [19]. Age of onset typically occurs prior to the age of 30 years, often coinciding with intensified body image concerns and exposure to social media and peer networks that valorise muscularity [20]. Although men remain the predominant consumers, AAS use among women, often centred around leanness, performance and therapeutic self‐optimisation, is an emerging phenomenon that remains understudied [16, 17].

Motivations for AAS use are multi‐factorial, encompassing aesthetic, performance, psychological and social drivers that differ across gender, age and social milieu. For some, AAS use reflects a deeply embodied pursuit of performance; for others, it represents a pragmatic strategy for mood enhancement, recovery or dissatisfaction with body image [10, 21]. For a subset of consumers, AAS use is closely intertwined with clinically significant body image pathology, including muscle dysmorphia [22], where AAS use may function as a form of self‐medication for other eating disorder pathologies [23]. These motivations shape not only the onset of use but also the preferences for drug type, delivery route and usage pattern.

Anabolic–androgenic steroids (AAS) are consumed through multiple delivery routes, including intramuscular or subcutaneous injection, oral ingestion and transdermal preparations (gels, creams, patches) [24, 25]. In non‐medical consumers of AAS, injectable esterified testosterone derivatives (e.g. testosterone, nandrolone) are common, followed by oral alkylated compounds such as oxandrolone and methandrostenolone [26]. Oral formulations are popular among novice consumers for their ease of use [27, 28]. Transdermal and subcutaneous testosterone preparations, commonly prescribed in clinical contexts [29, 30], are also used non‐medically, particularly among older men seeking vitality or libido enhancement [31]. Non‐medical consumers of AAS often employ ‘cyclic’ (i.e. a period of time on AAS followed by a period of time off AAS) or ‘blast and cruise’ (with periods of alternating low‐ and high‐dose use of AAS) approaches [11]. These pharmacological and behavioural variations reflect not only differing levels of expertise but also distinct social identities and relationships to risk.

Recent typological work [32] has been central to understanding these trajectories, identifying and suggesting four types of AAS consumer: the expert type, the wellbeing type, the you‐only‐live‐once type and the athlete type. The four types are developed around two overarching categories, namely the consumer approach to *risk* and *effectiveness*. However, two highly prevalent groups warrant explicit consideration within typological frameworks: cisgender gay and bisexual men, and individuals using AAS in the context of muscle dysmorphia. Cisgender gay and bisexual men disproportionately report AAS use, often in relation to sexualised body ideals, community‐specific norms around muscularity, and the social capital attached to size and leanness [19]. Use within this group may overlap with existing typologies (e.g. image‐driven use) but is also shaped by distinct social contexts and engagement with sexual health services, suggesting a partially differentiated consumer profile [33]. Similarly, self‐medicating individuals with muscle dysmorphia may traverse multiple typological categories over time, with use driven by attempts to alleviate persistent body‐related distress [23]. These groups also differ in product choice and routes of administration, with implications for both short‐ and long‐term harm. Evidence suggests that expert or long‐term consumers favour injectable AAS and polypharmacy, whereas newer, image‐driven consumers more often initiate with oral agents [34]. For gay and bisexual men, dosing and compound selection may additionally reflect pressures to rapidly modify or sustain appearance [19]. Overall, AAS use patterns are shaped not only by pharmacology but by socially situated values, identities and relationships to risk.

Long‐term use frequently progresses into AAS dependence, a recognised but under‐acknowledged substance use disorder [14, 35]. Meta‐analytic evidence estimates that approximately one‐third of long‐term AAS consumers meet the criteria for dependence [15], identifying predictors such as prolonged use duration, higher cumulative doses and co‐use of ancillary drugs (e.g. stimulants, growth hormone) [36]. Among individuals using AAS in the context of muscle dysmorphia, dependence trajectories may be particularly entrenched, with cycles driven by psychological distress [37]. Together, these findings underscore that while some AAS consumers engage in temporally limited or experimental use, a substantial subset transitions to chronic, dependence‐like patterns that carry significant physiological and psychosocial consequences. Importantly, pharmacological differences between oral, injectable, and transdermal preparations create distinct acute and chronic risk profiles. Recognising these differences is essential for moving harm reduction beyond generic guidance, and for examining how risks vary across delivery methods and patterns of use.

## (DE)CONSTRUCTING DRUG HARMS: ACUTE VERSUS CHRONIC RISK ACROSS ADMINISTRATION ROUTES

Before examining AAS, it is essential to contextualise risk as a product of the drug being used and the route of administration across psychoactive substances. Injection has historically been framed as having the highest risk owing to rapid bioavailability, overdose potential and BBVs or bacterial infections [38]. From the mid‐20th century, injecting heroin and methamphetamine was associated with increased morbidity, forming the basis for harm reduction messaging that positioned oral and inhalation routes as safer [39]. Community health services like needle and syringe programmes (NSPs) institutionalised this framing in the 1980s, emphasising sterile equipment and aseptic technique, while illustrating that risk is context dependent [2, 40]. For AAS, injection outside clinical supervision raises concerns about contamination, self‐taught technique and stigma, rather than acute toxicity or overdose [21]. Chronic harms associated with AAS use are physiological [9, 41], including cardiovascular disease, hepatic and renal dysfunction, and endocrine disruption [42, 43], as well as psychosocial, with harms such as mood disturbance and body image distress [44, 45, 46]. Recognising these nuances is essential for developing evidence‐informed, pragmatic harm reduction tailored to AAS consumers.

In harm reduction discourse, it is increasingly important to distinguish not only between acute (e.g. overdose) and chronic (e.g. BBV) harms, but also between the sources of harm: those stemming from the pharmacological properties of a drug (e.g. opioids) and those arising from the route of administration (e.g. injection). For example, with opioids there are dominant acute harms stemming from the pharmacological properties of the drug: respiratory depression leading to overdose, which is potentiated by a toxic drug supply (such as fentanyl/nitazenes) that can cause fatal outcomes [47, 48, 49]. Acute harms can also derive from the route of administration: for instance, sepsis or local abscesses may occur rapidly when non‐sterile injection technique is used [50, 51]. In contrast, chronic harms may also accumulate from both the drug and its administration route. For opioids, chronic pharmacological harms include potential cardiometabolic effects or neurological sequelae associated with long‐term use [52]. Importantly, chronic harms also encompass the development of opioid dependence or opioid use disorder, an escalating condition characterised by tolerance, withdrawal and compulsive use, which amplifies vulnerability to both pharmacological harms (e.g. overdose risk during relapse or periods of fluctuating tolerance) and route‐related harms, by increasing the frequency and intensity of injecting [52, 53]. Chronic harms attributable to the route of administration are well documented among people who inject opioids, and include: BBVs, such as HIV or hepatitis C (HCV); bacterial infections (e.g. cellulitis or endocarditis); and other injection‐related complications [51, 52, 54]. Harm reduction interventions have therefore focused on mitigating these acute and chronic risks through sterile equipment and injection education via NSPs [2], naloxone distribution [55], and standalone or combined drug checking and supervised consumption facilities [56, 57].

This dual‐axis perspective (drug vs route × acute vs chronic) helps clarify why AAS present a fundamentally different risk profile. Regarding acute harms associated with the drug, AAS are not associated with life‐threatening acute overdose, with the exception of adjacent enhancement drugs like insulin when used non‐medically [8]. Regarding acute harms associated with the route, there is the potential for acute injection‐related complications (e.g. abscesses) [58, 59, 60]. In terms of the chronic harms, the primary risks of AAS are physiological harms arising from long‐term exposure, as is common in AAS dependence (e.g. cardiovascular, hepatic, renal, endocrine harms) [9]. In terms of chronic harms associated with the drugs, oral AAS differ substantially from injectable AAS in their harm profiles. First‐pass hepatic metabolism produces toxic metabolites capable of cholestasis, peliosis hepatis, renal strain and, in prolonged use, hepatic carcinoma [61, 62]. Products in the oral AAS market are commonly falsified, as demonstrated by frequent mislabelling or adulteration, increasing the risk beyond that of regulated therapeutic products [63, 64]. Further, transdermal AAS creams are effective at bypassing the route of administration risks; however, they have the potential for transference to partners and children [30]. Table 1 summarises selected AAS compounds, typical administration routes, key acute and long‐term harms and representative consumer profiles. Examining these differences allows for more accurate comparisons of administration routes across substances, and the adaptation of harm reduction strategies to unique pharmacological and temporal AAS profiles.

**TABLE 1:** Example AAS compounds by category, route, acute and chronic harms, and typical consumer.

Category	Compound	Usual route	Acute harms	Chronic harms	Common consumer profile
Medically used AAS (injectable, transdermal, oral)	Testosterone esters (enanthate, cypionate), transdermal testosterone gels or creams	IM or subcutaneous [65], transdermal [30]	Injection‐related acute events: injection‐site abscess/cellulitis; pulmonary oil microembolism [66]	Endocrine suppression, dyslipidaemia, erythrocytosis, infertility and elevated cardiovascular disease risk [25, 67], variable absorption and transfer risk to partners or children [30]	Both initiates and experienced male consumers [68]; consumers avoiding injections [30, 69]
	Oxandrolone (Anavar)	Oral [70]	Acute hepatic injury [71]	Hepatotoxicity, renal strain, dyslipidaemia [71]	Women seeking ‘milder’ and less virilising AAS [17, 72]
Veterinary injectable preparations	Trenbolone (acetate or enanthate)	IM	Acute neuropsychiatric events; injection‐related acute events [73, 74]	Marked dyslipidaemia, hypertension, arrhythmia risk and pronounced psychological effects [26, 74, 75]	Consumers prioritising rapid physique change [26, 73]
	Boldenone undecylenate (Equipoise)	IM	Injection‐related acute events [66]	Polycythaemia, sustained blood pressure elevation and cardiovascular strain [76, 77]	Strength and physique consumers seeking mass and appetite increase [78]
Non‐clinical oral preparations	Methasterone (Superdrol)	Oral	Acute hepatic injury [79]	Extreme hepatotoxicity and profound dyslipidaemia [79, 80]	Young male consumers drawn to orals; experienced male consumers [81]
	Chloro‐dehydro‐methyl‐testosterone (Turinabol)	Oral	Acute injury—leiomyosarcoma [82]	Severe hepatotoxicity, dyslipidaemia, infertility, cancer [82, 83]	Young consumers drawn to orals [82]

*Note*: Categories distinguish pharmaceutical human‐use androgens, veterinary preparations repurposed for physique enhancement, and oral AAS. Routes are listed as commonly used among non‐medical consumers as well as approved clinical routes. Harms reflect human clinical studies, cohort data, and case reports where available; veterinary preparations and oral AAS rely more heavily on case‐based evidence. Consumer profiles are indicative patterns, not prescriptive categories.

Abbreviations: AAS = anabolic–androgenic steroids; IM = intramuscular.

## LEARNING ACROSS DRUGS OF DEPENDENCE: EXPANDING HARM REDUCTION

Lessons from established harm reduction models for other drugs of dependence can inform strategies for AAS consumers. First, we consider interventions related to route of administration, and how these can be augmented through the integration of acute and chronic harm management. Injecting AAS does carry specific risks. BBVs such as HIV and HCV are less prevalent among people who inject AAS than among people who inject opioids or stimulants. For example, in an Australian cohort of 156 people who inject AAS, the HIV prevalence was 1% and the HCV prevalence was 9% [84]. However, recent work suggests that for some AAS‐using subgroups, particularly gay and bisexual men, HIV and BBV risk may be driven less by injecting practices alone and more by intersecting sexual and drug‐use behaviours [85]. Studies among sexual minority men who use AAS show higher rates of condomless anal intercourse, increased numbers of sexual partners and elevated use of psychoactive substances, such as methamphetamine and gamma hydroxybutyrate [86]. This helps explain why harm reduction approaches focused narrowly on BBV prevention through the provision of injecting equipment may miss broader infection risks and health priorities among people who inject AAS. At the same time, injection‐related morbidity remains common. In Australian cohorts of people who inject AAS, approximately 20% report abscesses or other injection‐site infections [87], while UK data indicate that 36% report injection‐related infections within a 12‐month period [88]. Unsafe practices contribute to these outcomes, including sharing multi‐use vials, reusing needles or containers, and the occasional use of the injecting equipment of others, all reported in Australian and UK studies (approx. 14–29%) [60, 84]. Because a large proportion of AAS consumers inject (>70%) [63], route‐of‐administration interventions remain a critical touchpoint for harm reduction. However, these interventions must extend beyond BBV prevention alone to address the broader constellation of injection‐related, sexual and long‐term health risks that shape AAS use.

Augmenting injection‐related interventions for AAS with structured harm management can draw directly on lessons from other drugs. Acute harms can be mitigated through targeted education on intramuscular and subcutaneous injection techniques, aseptic practices and single‐use equipment. Chronic harms can be addressed by linking consumers with health services for the monitoring of organ function, such as liver fibrosis scans or cardiovascular screening, analogous to long‐term follow‐up in people with HCV, as seen in other communities of people who inject drugs [89]. Psychosocial harms can be considered through relational care and peer‐led support programmes, providing guidance, shared experience and ongoing engagement to explore community preferences, as seen in harm reduction initiatives for other people who use drugs [90, 91]. In parallel, harm reduction services engaging people who use AAS, particularly gay and bisexual men, should incorporate condom promotion, routine sexual health screening and education about pre‐exposure prophylaxis (PrEP) as core components of care. Rather than treating injecting as the sole or primary site of infection risk, harm reduction for AAS must recognise the convergence of injecting, sexual behaviour and polysubstance use in specific populations. This approach maintains fidelity to established harm reduction principles while adapting them to be relevant to people who use AAS. However, interventions focused on route of administration alone cannot address a major driver of harm: dosing practices. As AAS use typically involves structured, long‐term regimens rather than episodic consumption, risk is shaped primarily by substance and dosage selection.

Second, we consider interventions targeting substance quality and composition to reduce both acute and chronic risks. These risks apply across routes of administration, as illicit AAS are produced within unregulated supply chains. Many consumers use oral AAS alongside injectables, while others rely exclusively on oral compounds, making contamination, mislabelling and potency variability central harm drivers, even in the absence of injection‐related risk. Although there is clinical infrastructure to support the medical use of AAS [92, 93, 94, 95, 96, 97], non‐medical AAS consumers rely on illicit markets where products may be mislabelled or contaminated [98], and where supply chains are further disrupted by forensic seizures that remove products before they reach consumers [99]. These systemic conditions directly shape patterns of harm and intersect with the structural challenges clinicians report [100, 101], including uncertainty about product content, inconsistent dosing and limited evidence‐based guidelines, when attempting to assess, monitor or treat this cohort. Even well‐designed injection or dosing interventions rely on ensuring that consumers know what they are using, as unstandardised products remain a key driver of harm.

Drug checking has become indispensable in other drug settings, such as for opioids, especially with potent adulterants like fentanyl and nitazenes, because it provides chemical verification, alerts consumers to dangerous contaminants and supports safer‐use decisions [57, 102]. For AAS, similar services are emerging: in Zurich, Switzerland, a pilot drug‐checking service analysed 71 consumer‐provided AAS samples via gas chromatography–mass spectrometry (GC–MS), finding that over half were falsified, while reporting very high client satisfaction [103]. In Australia, mixed‐method research from a Queensland‐based testing trial analysed 58 community‐submitted AAS samples and found significant purity and identity discrepancies (>50%); follow‐up interviews with submitters as well as non‐submitters who viewed the testing results showed that receiving this chemical feedback led to more cautious use behaviours [12]. By integrating drug checking with harm reduction infrastructure for AAS, consumers can make more informed choices, reduce exposure to harmful or fake substances and engage with low‐threshold services. This suggests AAS‐integrated drug checking could reduce risk directly and serve as a gateway to broader health care, including counselling, monitoring or referral when dangerous compounds are detected.

Within this broadened model, dosing and compound selection practices represent a key area for integration. Messages such as ‘start low, go slow’ are widely used in harm reduction for other drugs, yet they translate imperfectly to AAS because AAS use is rarely episodic. Unlike substances where individuals may escalate their dose incrementally in response to acute effects, AAS use typically involves pre‐planned, long‐term regimens in which target dosages, cycle length and ancillary drugs are determined in advance, often before use begins. In therapeutic contexts, intramuscular, subcutaneous or transdermal testosterone is typically administered at dosages ranging from 50 to 200 mg per week, carefully titrated and monitored to restore physiological androgen levels in hypogonadal men [92, 93, 94, 95, 96, 97]. In contrast, community practices often involve supraphysiological regimens of 250–1500 mg per week, far exceeding these thresholds [26]. Contextually relevant harm reduction for AAS should therefore prioritise reducing harms associated with high‐dose cycling by providing evidence‐informed guidance on dosage ranges, half‐lives and pharmacokinetics, alongside information on compounds with comparatively lower risk profiles (e.g. testosterone). These lessons highlight practical pathways for harm reduction tailored to AAS, with implications for services, workforce and policy.

## IMPLICATIONS AND NEXT STEPS

First, harm reduction for AAS should equip consumers with practical, accessible tools to minimise harms. E‐health bundles targeting newer consumers can meet them where they are, providing guidance on safer use, drug checking [57], and aligning dosage and compound selection with clinical regimens [57, 94, 95, 96, 97]. Consistent with recently proposed prevention frameworks that conceptualise AAS harm reduction in terms of primary, secondary and tertiary prevention [104], digital bundles can provide structured guidance across these layers: primary prevention, by avoiding high‐risk compounds (e.g. trenbolone) and unsafe practices (e.g. dosing regimens); secondary prevention, through self‐directed monitoring (e.g. blood pressure checks, liver and kidney panels); and tertiary prevention, by offering evidence‐informed information on safer self‐management practices (e.g. guidance on the cautious use of ancillary medications such as aromatase inhibitors and statins). Digital health bundles offer a practical way to deliver these insights to contextually appropriate places that AAS consumers frequent, such as forums and social media [105, 106].

Second, workforce development and health service enhancement are needed. Community health services and initiatives, which are known to have frequent contact with this cohort (e.g. NSPs) [107, 108], need to be equipped to provide accurate, AAS‐specific education for people who use AAS medically, non‐medically and those who are AAS dependent. Although many NSPs already distribute injecting equipment, few offer guidance tailored to intramuscular or subcutaneous administration, multi‐use vials, site rotation or managing slow‐developing injection‐site infections [11]. Given the evidence of overlapping sexual and injection‐related risks among some AAS consumers, particularly cisgender gay and bisexual men [86], services should also be prepared to offer or link clients to sexual health resources, including condoms, PrEP referral or prescribing pathways, and routine HIV and hepatitis screening. Some AAS (e.g. trenbolone) exhibit potent psychoactive effects that shape mood, cognition and behaviour [73]. Further, long‐term AAS use can lead to dependence syndromes marked by physiological adaptation and psychological attachment [14, 109]. Screening for muscle dysmorphia and related body image pathology represents an additional, underutilised intervention point, with referral pathways for psychological support where indicated. Expanding harm reduction in this context requires addressing both acute harms (abscesses, cellulitis, sepsis) and chronic harms (left ventricular hypertrophy, dyslipidaemia, erythrocytosis, mood), supporting safe injection, and monitoring emerging risks through objective measures (blood tests evoking liver and kidney markers, lipid panels, haematocrit and, also where possible, echocardiography) [110, 111], alongside subjective indicators such as mood and relationship strain [26, 73]. Workforce development should also normalise collaboration with peer educators, who currently provide much of the technical and experiential knowledge for consumers [11].

Third, these strategies point to a broader conceptual shift in harm reduction. This shift requires coordination across researchers, clinicians, harm reduction workers and peers through shared frameworks, language and care pathways. Integrating AAS expands the understanding of drug‐related harm to include long‐term, cumulative and pharmacologically distinct risks, while recognising the diversity of people who use drugs for enhancement, aesthetic or performance purposes. Doing so reinforces harm reduction as an approach that must remain flexible, evidence‐informed and responsive to lived experience.

## CONCLUSION

The use of AAS presents a fundamentally different risk profile from the acute, event‐based harms that have shaped mainstream harm reduction. Expanding harm reduction to include AAS requires approaches that address both acute and chronic physiological harms, as well as psychosocial, sexual health and supply‐related risks, in ways that are accessible and contextually relevant for consumers. Digital health interventions with integrated AAS checking data can directly support consumers in making safer choices, while workforce development ensures that services can provide tailored (and peer‐led) education and clinical guidance through monitoring, sexual health and body image pathology screening. Embedding AAS into existing harm reduction frameworks also broadens the conceptual scope of interventions, recognising the diversity of people who use drugs and the variety of goals, motivations and patterns of use.

## AUTHOR CONTRIBUTIONS

**Timothy Piatkowski:** Conceptualization (lead); writing—original draft (lead); writing—review and editing (lead). **Jason M. Nagata:** Writing—review and editing (equal). **Suzanne Nielsen:** Writing—review and editing (equal).

## DECLARATION OF INTERESTS

No competing interests to declare.

## Data Availability

Data sharing not applicable.
